# Survival and Predictors in Patients With Breast Cancer Undergoing Surgery for Spinal Metastases: Insights From a Single‐Center Cohort

**DOI:** 10.1155/ijbc/7130498

**Published:** 2026-08-10

**Authors:** Leonardo Olijnyk, Vivian Huong Hoang Thien Le, Edisond Florial, Fidaa Al-Shakfa, Jean-Marc Mac-Thiong, Maroun Rizkallah, Ghassan Boubez, Jesse Shen, Zhi Wang

**Affiliations:** ^1^ Department of Neurosurgery, Saint John Regional Hospital, Saint John, Canada, horizonnb.ca; ^2^ Faculty of Medicine, University of Montreal, Montreal, Canada, umontreal.ca; ^3^ Centre de Recherche du Centre Hospitalier de L′Universite de Montreal, Montreal, Canada; ^4^ Department of Surgery, Hôpital du Sacre-Coeur, Montréal, Quebec, Canada; ^5^ Department of Orthopedic Surgery, Dr. Georges-L.-Dumont University Hospital, Moncton, Canada; ^6^ Department of Surgery, Division of Orthopedic Surgery, Centre Hospitalier de L′Universite de Montreal, Montreal, Canada

**Keywords:** breast cancer, oncology spine surgery, prognostic factors, spinal metastases

## Abstract

**Background:**

Breast cancer is the most commonly diagnosed malignancy among women globally, with spinal metastases significantly impacting morbidity and mortality. Surgical intervention plays a crucial role in managing symptomatic spinal lesions, yet prognostic factors and outcomes remain areas of active investigation. This study aims to evaluate survival rates and identify predictors in patients with breast cancer undergoing surgery for spinal metastases.

**Methods:**

A retrospective analysis was conducted on 97 patients who underwent surgery for spinal breast cancer metastases at a single center over a 12‐year period. Data included epidemiological and preoperative profiles, biological markers, and radiological findings. Survival outcomes were assessed using Kaplan–Meier analysis, and prognostic factors were analyzed via Cox regression.

**Results:**

Postoperative overall survival rates were 72%, 64%, and 34% at 1, 2, and 5 years, respectively. Significant predictors of survival included tobacco use (HR 1.7, *p* = 0.025), estrogen receptor (HR 0.224, *p* = 0.03), triple‐negative subtype (HR 4.1, *p* < 0.01), and lower Karnofsky scores (HR 0.488, *p* < 0.01). Spinal instability and preoperative neurological status did not significantly influence survival. Complications occurred in 17% of patients, with no 30‐day mortality.

**Conclusion:**

This study provides one of the largest single‐center experiences of surgery for spinal metastases from breast cancer in the modern treatment era. We found that overall preoperative performance and biological markers, remains a key predictor of survival, while neurological status, disease spread and spinal instability have no impact than in long‐term survival. These results reflect advances in systemic therapy and surgical care, supporting surgery as an important part of multidisciplinary management for selected patients.

**Key Points**

‐Aggressive surgical treatment in spinal metastatic breast cancer should always be considered nowadays.

‐Even patients with advanced disease likely benefit from operation for symptomatic spinal lesions.

‐Multidisciplinary approach for this population is essential.

## 1. Introduction

Breast cancer is the most frequently diagnosed cancer in women worldwide. Despite reports of decreasing mortality, breast cancer continues to be one of the leading causes of mortality related to malignancy [1, 2]. By the time of diagnosis, metastatic disease is present in 6%–10% of patients, but it is responsible for roughly two‐thirds of diagnosed metastases and up to 75% of patients will develop skeletal metastases during a spread disease [3–5]. The spine is the most prevalent site of bone metastases, and up to one‐third of these lesions become symptomatic [5–8]. In patients with breast cancer metastases (BCM), the condition is considered incurable. The treatment goals should aim to prolong life, preserving function and quality of life [9, 10].

Nevertheless, these patients are experiencing increased longevity due to significant advancements in systemic therapies developed in recent decades. Tailored treatment to molecular subtypes, along with novel generation of chemotherapy, has improved survival in patients with breast cancer [11, 12]. Early detection in screening programs plays important role but patients diagnosed with advanced disease can also have an optimistic survival such is the progress of pharmacotherapy [13]. As a consequence, more patients are expected to develop symptomatic spinal metastases, broadening the indications for spine surgery. This evolution has driven research efforts aimed at defining prognostic and predictive factors that inform surgical eligibility and the choice of treatment modalities in breast cancer spinal metastases [14, 15].

In this context, several aspects have been investigated regarding assessment, operative strategy, and prognosis following surgery for BCM [15, 16]. Histopathological profile, biological markers, preoperative functional status, precise radiological evaluation, extension of bone disease, and presence of visceral tumor, among others, have been examined to determine their influence on outcomes in spine surgery for breast cancer [17, 18]. These new data have encouraged multidisciplinary teams to adopt more aggressive approaches in the management of spinal BCM [19]. Nowadays, regardless of the palliative condition in some patients, surgery increases longevity and has the potential to preserve quality of life by preventing neurological deterioration, maintaining spinal stability, and offering better chances to control local disease with radiotherapy [20].

In this study, we report the experience of 103 surgeries at a single academic medical center for 97 patients with spinal BCM over a 12‐year period. We performed a retrospective chart review from January 2011 to October 2023 to analyze the epidemiological profile, preoperative characteristics, radiological findings, exposure to adjuvant therapies, and survival of patients who underwent surgery with or without spine fixation. In addition, we investigated the impact on life expectancy of spinal instability, oncological history, prevalence of metastases, and performance condition.

## 2. Method

### 2.1. Study Population and Selection Criteria

We performed a retrospective review of all patients that received surgical intervention for metastatic breast cancer lesions in the spine from January 2011 to October 2023 at our institution. The selection criteria for all subjects included histological confirmation of disease, surgery performed by one of the five fellowship‐trained spine surgeons from the orthopedic or neurosurgery department, available preoperative storage imaging, and available clinical information. The requirement for informed consent was waived, as the data utilized in the study were anonymized and contained no identifiable patient information. The data were collected from a digital chart, and the project protocol was submitted and approved by the institutional ethics committee under registration #2025‐12446.

Surgical indication was determined after joint decision with the oncology team. Patients undergoing operation met one or more of the following criteria: spinal cord or nerve root compression with neurological compromise; evidence of radiographic spine instability due to the lesion or spine Surgical Instability Neoplastic Score (SINS) [21] ≥ 7; tumor progression despite systemic treatment or/and radiotherapy; or intractable pain. All patients were medically stable, understood and consented to surgery, and had a life expectancy longer than 3 months. The length of fixation, number of laminectomies, surgical corpectomy, anterior column reconstruction, approach (anterior, posterior or both), and use of bone graft were individually determined by the surgeon.

### 2.2. Data Collection and Parameters

The following variables were retrieved from the medical records: demographics, tobacco use, preoperative neurological condition (graduated according to the Frankel score), performance status (Karnofsky scale), time of diagnosis, length of hospital stay, anatomic level operated, history of radiotherapy/chemotherapy before operation, adjuvant therapy, overall survival (OS) time, radiographic information, presence of oncological systemic disease, peri and postoperative complications, and genetic analysis for ER/PR receptors and HER2 status.

Neurological status was considered in last physical examination before surgery, which was preferentially performed by the surgeon. Only neurologically intact patients and with no walk disturbance that could be caused by tumor compression were considered Frankel E. Radiographic assessment was based on CT, MRI, or both. The location level involved in the disease was considered as follows: cervical, C1–C6; cervicothoracic, C7–T1; thoracic T2–T11; thoracolumbar, T12–L1; lumbar L2–L4; lumbosacral L5–S1; sacral S2–S5. Any multilevel disease that included at least one vertebra at a junctional location was classified as such.

Patients were considered to have a new diagnosis of breast cancer if they have never had a malignant lesion identified by histological tissue in the breast. Patients who underwent biopsy or any other or histological examination prior to spinal surgery but this was already in work‐up investigation for the symptomatic spine lesion with an indication for surgery, were also considered as having a new diagnosis of breast cancer.

### 2.3. Surgical Technique

Surgical strategies—including decompression alone, corpectomy, and instrumented fusion—were tailored according to the individual patient’s pathology and clinical condition, at the discretion of the treating surgeon. The guiding principle was to balance operation morbidity with procedural efficacy, with the overarching goal of enabling patients to continue systemic and radiotherapy treatments as promptly as possible. Generally, we recommended operative treatment for patients with life expectancy longer than 3, if they were suitable for the anesthetic time. Neurological decompression was always a surgical priority. Local adjuvant radiotherapy, with total dose delivered of 20–24 Gy, was administered a few weeks after surgery, fractionated according to institutional oncological protocols.

### 2.4. Statistical Analysis

Postoperative OS was estimated using the Kaplan–Meier method. Variables were coded yes/no for tobacco use, ER, PR, triple‐negative tumors (negative for ER, PR, and HER2 markers), presence of visceral metastasis, adjuvant chemotherapy and radiotherapy. Other variables were coded binary or ternary as follows: age, ≤ 60 versus > 60; Frankel, E versus A–D; KPS, 100–80 (able to perform normal activities) versus 70–10 (unable to perform daily regular activities with varying need for assistance); bone metastases, non versus 1–2 versus ≥3; spine metastases, 1 versus 2 versus ≥3; SINS, 0–6 versus 7–12 versus ≥13. Differences in survival between subgroups were assessed using the log‐rank test in univariate analysis. Prognostic variables that reached statistical significance in univariate testing were subsequently entered into a multivariate model using Cox proportional hazards regression to identify independent predictors of survival. A *p* value < 0.05 was considered statistically significant. All analyses were conducted using SPSS software (Version 21.0.0; SPSS Inc., Chicago, IL).

## 3. Results

Ninety‐seven patients were included in the review. One hundred and three operations were performed. Five patients underwent re‐operation for local recurrence, and one had a second surgery at a different site. The demographic, clinical characteristics of the cohort and distribution of lesions are summarized in Table 1.

**Table 1:** Demographics and clinical characteristics.

	*N*(%)
Gender	
Female	96 (99%)
Male	1 (1%)
Age (years)	
Mean/median (range)	58.4/60 (27–83)
New diagnosis at time of spine surgery	19/94 (20%)
Smoking history	14/94 (15%)
Hormone receptor status	
Estrogen positive	78/97 (80%)
Progesterone positive	60/97 (62%)
Triple‐negative	9/91 (10%)
Preoperative neurological status (Frankel score)	
Frankel E	45/95 (47%)
Frankel D	47/95 (49%)
Frankel C	3/95 (3%)
Adjuvant therapy prior to surgery	
Radiotherapy	59/94 (63%)
Chemotherapy	72/94 (77%)
Spinal level of lesion	
Cervical (C1–C6)	10/97 (10%)
Cervicothoracic (C7–T1)	9/97 (9%)
Thoracic (T2–T11)	50/97 (52%)
Thoracolumbar (T12–L1)	8/97 (8%)
Lumbar (L2–L4)	12/97 (12%)
Lumbosacral (L5–S1)	8/97 (8%)
Sacral (S2–S5)	0/97 (0%)
Spinal instability (SINS score)	
SINS 7–12 (Potentially unstable)	40/97 (41%)
SINS > 12 (Unstable)	57/97 (59%)
Karnofsky Performance Scale (KPS)	
10–70	43/93 (46%)
80–100	50/93 (54%)
Bone metastases outside the spine	
None	36/93 (39%)
1–2 Lesions	24/93 (26%)
≥ 3 Lesions	33/93 (35%)
Number of vertebral spine metastases	
1	14/93 (15%)
2	11/93 (12%)
≥ 3	68/93 (73%)
Visceral metastases present	69/93 (74%)

The mean age of the population was 58.4 (range: 27–83). There was only one man and 14 patients (15%) were smokers by the time of BCM diagnosis. The majority of patients were already known for breast cancer but 19 (20%) presented with the operative spine lesion with no prior history of oncological diagnosis. Prior to surgery, 63% and 77% of patients have received radiotherapy for the spine and chemotherapy, respectively. Those with previous radiotherapy had increased survival.

Preoperatively, 45 (47%) patients were neurologically intact, and 50 (54%) performed 80–100 on the Karnofsky Performance Scale (KPS). Considering the biological subtypes, 80% and 62% of the patients were positive for estrogen and progesterone, respectively. Triple‐negative patients stand for 10% of total. The thoracic level was the site more often involved, accounting for 50 occasions, whereas 10 patients had cervical lesions and the other nine had cervicothoracic surgical lesions. Eight patients were treated for thoracolumbar lesions, 12 for lumbar levels and the other eight for lumbosacral. No patient had purely sacral tumor. The location of the lesion did not influence OS.

### 3.1. Survival and Significant Prognostic Variables

**Figure 1:**
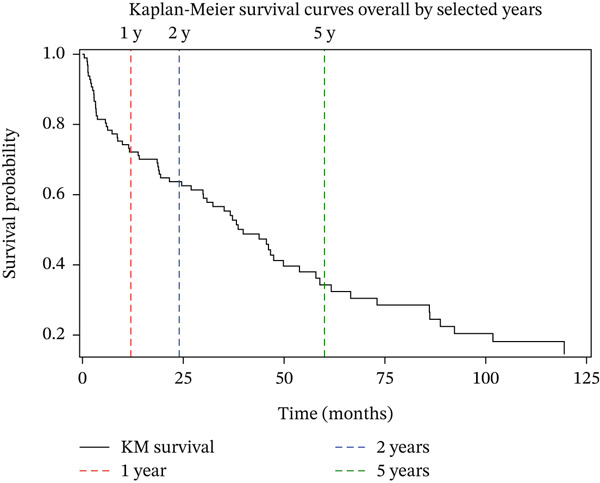
Postoperative overall survival in Kaplan–Meier curve. Kaplan–Meier curve demonstrating postoperative overall survival for the entire cohort (*n* = 97). Estimated survival rates were 72% at 1 year, 64% at 2 years, and 34% at 5 years following surgery. Censored observations are indicated by tick marks. Overall survival was calculated from the date of spinal surgery to the date of death or last follow‐up.

The postoperative OS was 72%, 64%, and 34% at 1, 2, and 5 years, respectively (Figure 1). Multivariate analyses of our data evidenced that tobacco use (HR 1.7; CI 95% 1.1–2.1), presence of estrogen hormone receptor (HR 0.224; CI 95% 0.1–0.9), triple‐negative tumors (HR 4.1; CI 95% 1.9–9), and lower preoperative Karnofsky performance (HR 0.488; CI 95% 0.3–0.8) significantly impacted OS (*p* < 0.05). Larger number of extraspinal bone metastases and number of spinal levels with metastases showed a trend to affect survival but did not reach significance.

Forty patients presented with potential instability of the spine before surgery, while 59 were considered unstable. This accounted for 41% and 59%, respectively. The preoperative SINS score did not influence in the OS of the population. Data is summarized in Table 2.

**Table 2:** Impact of variables in the postoperative overall survival after Cox regression multivariate analysis.

	Hazard ratio	95% Cl	*p*
Age (≤ 60 vs. > 60)	0.486	0.8–1.5	0.48
Smoking	1.73	1.1–2.1	**0.025**
ER	0.224	0.1–0.9	**0.03**
PR	0.199	0.5–1.5	0.65
Triple negative	4.1	1.9–9	**< 0.01**
Adjuvant chemotherapy	2.424	0.9–1.9	0.12
Adjuvant radiotherapy	0.085	0.6–1.5	0.77
Frankel (E vs. A–D)	1.207	0.7–1.5	0.27
SINS (7–12 vs. ≥13)	1.211	0.8–1.7	0.27
KPS (10–70 vs. 80–100)	0.488	0.3–0.8	**< 0.01**
Bone metastases			
Non vs. 1–2 vs. ≥3	1.42	0.9–1.8	0.08
Spine metastases			
1 vs. 2 vs. ≥3	1.347	0.9–2.0	0.07
Visceral metastases			
(Presence vs. absence)	2.4	0.7–8.69	0.87

*Note:* Values in bold are statistically significant (*p* < 0.05).

### 3.2. Operative Results

In our series, 18 patients (17%) had postoperative complications during hospitalization or within 30 days after surgery, four of which were due to infection related to the surgical site. There was no early death. Another three were readmitted later than 30 days with complications related to the surgical site. Two of them experienced failure of instrumentation and wound infection concomitantly. The mean length of stay after surgery was 11.8 days, with a median of 8. Data is resumed in Table 3.

**Table 3:** Operative data.

Duration of surgery (minutes)	Mean: 191.3/Median: 167
Estimated blood loss (mL)	Mean: 905
Length of postoperative stay (days)	Mean: 11.8/Median: 8
30‐Day postoperative complications	18 patients (17%)
Surgical site infections (within 30 days)	4 cases
Instrument failure and late infections	2 cases
30‐Day mortality	None

## 4. Discussion

Breast cancer incidence has been increasing in the Western countries, accompanied by a modest decline in mortality rates. This trend reflects the expansion of population screening programs, along with advances in systemic therapies [2, 22]. As patients live longer, there is a corresponding rise in the prevalence of metastatic bone disease [23, 24]. Nevertheless, the presence of bone BCM is significantly associated with a decline in quality of life and reduced survival [25, 26]. For patients with spinal BCM, it is well established that surgical intervention is beneficial in situations of progressive neurological deficit, spinal instability, marked cord compression, or tumor progression despite radiotherapy and pharmacologic treatment [27]. Given this context, it is anticipated that metastatic spine disease will become increasingly common in the routine practice of spine surgeons. In the present study, 80% of patients who underwent surgery had a prior history of breast cancer, with their condition progressing to spinal metastatic disease, and making them candidates for spinal surgery.

In 2013, Zadnik et al. [19] reported a median survival of 27 months following surgery for spinal BCM. This represented a 28% increase compared with a similar operative cohort shown by the same group 6 years earlier [7]. The authors attributed this improvement to the impact of the new adjuvant therapies, which became more widely available from the first to the second study. Despite the heterogeneous behavior of breast cancer, individuals with metastatic disease have better prognoses than those with other types of malignancies. In our cohort, the mean survival time was 72% at 1 year, 64% at 2 years, and 34% at 5 years, consistent with retrospective reviews published in recent years, confirming a more favorable prognosis when compared with groups operated on for spinal lesions of lung cancer and melanoma, which are known to be often refractory to systemic treatment [28–30]. Palliative surgery, which is aimed at local control of spinal lesions, can improve quality of life by relieving pain and potentially reversing neurological deficits. The prolonged survival observed in patients with BCM justifies aggressive surgical intervention and requires a comprehensive multidisciplinary approach to ensure a good physical and mental evolution in the postoperative phase.

Several studies in the literature have reported postoperative survival rates in patients with breast cancer undergoing surgery for spinal metastases, providing a useful benchmark for comparison. Amelot et al. observed 1‐year, 3‐year, and 5‐year survival rates of 83.2%, 54.3%, and 39.9%, respectively [14]. Similarly, Chan et al. reported survival rates of 89.2% at 1 year, 60.7% at 3 years, and 48.2% at 5 years [16]. Zhao et al. documented 1‐year, 3‐year, and 5‐year spinal metastasis‐free survival rates of 85.9%, 61.5%, and 35.9%, respectively [17]. In contrast, an earlier study by Zadnik et al. reported markedly lower survival outcomes, with a 1‐year OS of 66% and only 4% at 5 years, based on a cohort treated between 2002 and 2011 [19]. These variations likely reflect both improvements in systemic therapies over time and differences in patient selection criteria. Compared with these findings, our results align with more recent data, supporting the notion that patients with breast cancer with spinal metastases can achieve meaningful long‐term survival following surgery.

The investigation between preoperative neurological status and OS in surgical spinal BCM has been reported in several studies in the past years [14–16]. While some studies report a preoperative population with outstanding rates of patients neurologically compromised [17, 19], others present a surgical cohort vastly intact before surgery [14]. It reflects the heterogeneity among the cohort analyzed. Factors that may account for these interstudy differences include characteristics inherent to the population treated at a single center, cultural aspects, patients’ access to healthcare systems, and institutional practices regarding surgical triage. It may also potentially reflect the transition of the operative practice in patients with spinal BCM over the last decade.

**Figure 2:**
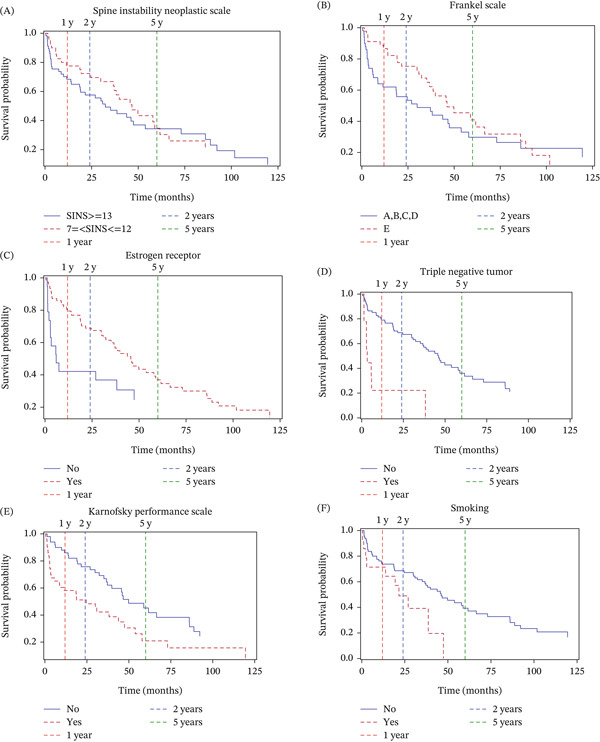
Analysis of different factors on the overall survival (A to F). Kaplan–Meier curves for (A) SINS score, (B) Frankel Scale, (C) Estrogen receptor, (D) Triple Negative, (E) KPS, and (F) Smoking. (A) Spine Instability Neoplastic Scale Spine Instability Neoplastic Score (SINS): potentially unstable (7–12) versus unstable (> 12). No significant difference in overall survival was observed between groups. (B) Frankel Scale Preoperative neurological status according to the Frankel scale: neurologically intact (Frankel E) versus neurologically impaired (Frankel A–D). Neurological status did not significantly influence long‐term overall. (C) Estrogen Receptor Estrogen receptor (ER) status: ER‐positive versus ER‐negative tumors. ER positivity was associated with significantly improved survival. (D) Triple Negative Tumor Triple‐negative subtype: triple‐negative versus non–triple‐negative tumors. Triple‐negative tumors were associated with significantly worse survival. (E) Karnofsky Performance Scale Karnofsky Performance Scale (KPS): 80–100 versus 10–70. Higher preoperative functional status was associated with improved survival. (F) Smoking status: smokers versus nonsmokers. Tobacco use was associated with significantly worse overall survival.

In this series, 53% of the patients who underwent surgery exhibited some degree of neurological impairment, which was not significantly associated with reduced OS in the multivariate analysis, but it was associated with a better outcome at 1 and 2 years postoperative time (Figure 2B). Although surgical response to decompression of neurological structures varies after an installed deficit, operation typically offers better chances of functional recovery compared with the alternative of radiotherapy. This study shows that neurological status influences outcomes in the early postoperative period; however, beyond 2 years after surgery, patients who were initially neurologically compromised appear to have a prognosis comparable with those who were intact. This convergence may be attributable to advances in radiotherapy and systemic therapies.

In our cohort, 20% of the patients were not known for breast cancer and became aware of the condition during the investigation of a symptomatic spinal tumor, or presented an operative lesion at the moment of the primary diagnosis. The finding of a new diagnosis of breast cancer did not result in a significantly worse prognosis. The literature is scarce regarding data on spine surgical treatment specifically in individuals with a new diagnosis of cancer. Nonetheless, despite the diagnosis of the disease in an advanced stage, the good outcomes observed in this group encourage the interventions necessary to restore quality of life, maintain spinal stability, and to prevent neurological damage before further radiotherapy or pharmacological treatment.

Meanwhile, the finding that 80% of patients who underwent surgery for spinal metastases from breast cancer had a pre‐existing oncological diagnosis offers valuable insights for elaborating future follow‐up protocols, refining spine disease screening, and guiding medical management following the detection of bone tumor. However, this study did not provide specific details regarding the patients’ protocols of radiotherapy and chemotherapy. Future research may shed additional light on bone lesions that have progressed despite optimized nonsurgical treatment, thereby contributing to the ongoing discussion about the indications and efficacy of surgical intervention for spinal tumors.

We confirmed the association between the biological profile of breast cancer and prognosis in spinal BCM [8] (Figure 2C,D). HER2, which is an important marker in the assessment and prognosis of breast cancer, was analyzed and mentioned in this article in the context of patients with triple‐negative profile. Patients, positive for ER, had longer OS, while triple‐negative individuals, known for suffering from a more aggressive disease, demonstrated worse prognoses in our series. This subtype of breast cancer has a marked course of chemoresistance, tumor recurrence, and high incidence of distant metastases. Conversely, patients with positive hormone markers benefit from target therapies and most often present a more indolent disease.

Although the behavior of breast cancer is influenced by the status of the tumor receptor [31, 32], the postoperative evolution of metastatic patients is often analyzed as a single group. How the distinct nature according to the genotype can shape the operative approach to bone and spinal BCM is still not clear. Currently, biological aspects of the tumor do not impact surgical strategy or technique. However, with the consolidation of individualized treatment for oncological patients, these parameters have the potential to play a role in the future in relation to surgical decisions such as the use of minimally invasive technique, the extent of tumor removal, and spinal fixation techniques.

It has been reported that patients with spinal metastasis and concurrent lung, liver, or brain metastases present a worse prognosis than bone disease exclusively [33]. In our cohort, the presence of visceral metastases did not affect the OS in a multivariate analysis (Table 2), which is in line with recent studies evaluating patients undergoing surgery or radiation for BCM [19, 34]. Historically, dissemination to internal organs has been considered in the assessment of candidates for surgery for spine metastases in most of neoplasms [35]. On the other hand, multiple bone lesions can be part of decision‐making in oncological patients and, in our cohort, there was a trend that the greater the number of spinal and extraspinal metastases, the shorter the survival. Again, future researches will able to clarify this aspect. Although identification of bone spread represents an adverse prognostic factor in breast cancer, it is notably radiosensitive, and the majority of patients operated on spinal BCM will undergo complementary treatment of spine lesions after surgery.

We examined whether the degree of spinal instability before surgery, measured by SINS score [21], influenced postoperative survival. The score, originally developed to guide treatment decisions based on mechanical stability, has become a standard tool among spine surgeons over the past decade. In our cohort, no patient presented with a SINS <7, which reflects appropriate surgical selection, as patients with small or asymptomatic lesions are typically managed nonoperatively. Consistent with prior studies, including the series by Zadnik et al. [**19** **]**, our analysis showed no significant association between SINS category (potentially unstable vs. unstable) and OS (Figure 2A). Although patients with higher scores often have more extensive disease, the absence of a survival difference reinforces that SINS should not be interpreted as a prognostic metric but rather as an aid for operative decision‐making. Advances in surgical fixation techniques, perioperative safety, and adjuvant radiotherapy likely contribute to mitigating the negative impact of mechanical instability on outcomes in this population.

The balance between the risks and benefits of any intervention for patients with metastatic disease is delicate. Surgery for spinal BCM is always palliative, and the morbidity associated with the procedure should not overweigh the goal of life prolongation with equal or better functional status. The primary objective of our study was not to analyze in detail the impact of specific operative techniques on survival (e.g., fusion, corpectomy, separation surgery, or decompression alone). Instead, our focus was to provide a comprehensive evaluation of prognostic factors in patients undergoing surgery for spinal metastases from breast cancer, once the clinical indication for operative management had been clearly established.

In our study, 17.5% of patients presented with medical complications within 30 days, of which four were related to surgical infection. There was no fatal complication in the first 30 postoperative days, which reflected a thorough preoperative workup and appropriate patient selection. Indication for surgery included not only spine instability as previously discussed but also spine cord or nerves compression, tumor progression despite radiotherapy, and intractable pain. Nevertheless, patients who are candidates for spinal metastasis surgery constitute a heterogeneous population, and careful patient selection is essential to avoid subjecting these vulnerable patients to superfluous procedures while ensuring a judicious allocation of resources toward effective treatments.

## 5. Limitations

Our study contains some limitations. As a retrospective review, it carries the inherent bias of such design. Secondly, while this is one of the largest series reported to date, the sample size is still modest (*N* = 97), and the data were collected between 2011 and 2023, a period during which immune and chemotherapy advanced rapidly. A patient diagnosed at the beginning of this timeframe may have been subjected to more restricted therapeutic options than a patient treated in recent years. Lastly, at the time of final data collection, some patients were still alive, which could impact the accuracy of the survival analysis.

## 6. Conclusion

This study represents one of the largest single‐center experiences with surgery for spinal metastases from breast cancer, reflecting real‐world practice in the modern era of targeted and systemic therapies. Our findings confirm that tumor biology remains the main driver of survival, with estrogen receptor positivity associated with longer outcomes and triple‐negative disease predicting poorer prognosis. Interestingly, traditional factors such as neurological status and degree of disease spread have not showed a strong influence on long‐term survival. This likely reflects improvements in surgical safety, fixation techniques, and coordinated oncologic care that have reduced the impact of these factors.

Equally important, we found that global performance status, measured by the Karnofsky score, continues to be a reliable predictor of survival, underscoring the importance of functional reserve at the time of surgery. Patients who presented with spinal metastasis as their first sign of breast cancer had outcomes similar to those with a prior diagnosis, suggesting that surgery remains worthwhile even at advanced initial presentation. Together, these results contribute to refining prognostic understanding and can help clinicians better counsel patients on realistic expectations and the potential benefits of surgery within a multidisciplinary treatment plan.

## Author Contributions

L.O. contributed to the study conceptualization, methodology, data collection, writing and editing. V.H.H.T.L. and E.F. contributed to data collection. F.A.S. contributed to data curation and statistical analysis. J.M.M.T. and M.R. contributed to the study revision, validation, and feedback. J.S. contributed to the study conceptualization, revision, and data interpretation. Z.W. contributed with conceptualization, senior supervision, critical revision, coordination of the project.

## Funding

No funding was received for this manuscript.

## Conflicts of Interest

The authors declare no conflicts of interest.

## Data Availability

The data that support the findings of this study are available on request from the corresponding author. The data are not publicly available due to privacy or ethical restrictions.
